# Mendelian Randomization and Transcriptome Analyses Reveal Important Roles for CEBPB and CX3CR1 in Osteoarthritis

**DOI:** 10.3390/bioengineering12090930

**Published:** 2025-08-29

**Authors:** Hui Gao, Xinling Gan, Jing He, Chengqi He

**Affiliations:** 1 Rehabilitation Medicine Center and Institute of Rehabilitation Medicine, West China Hospital, Sichuan University, Chengdu 610000, China; gaohui0201@wchscu.cn (H.G.); xinlinggg@wchscu.cn (X.G.); 2 Key Laboratory of Rehabilitation Medicine in Sichuan Province, Chengdu 610000, China

**Keywords:** chemokines, osteoarthritis, Mendelian randomization, network, potential biomarkers

## Abstract

**Background**: Chemokines play a pivotal role in the progression of osteoarthritis (OA), but their exact mechanisms remain unclear. This study aimed to identify potential chemokine-associated biomarkers and investigate their causal relationships with OA. **Methods**: Transcriptome and genome-wide association study (GWAS) data were obtained from public databases, while chemokine-related genes (CRGs) were sourced from the literature. Initially, CRGs were expanded, followed by Mendelian randomization (MR) analysis, differential expression analysis, machine learning, and receiver operating characteristic (ROC) curve plotting to identify potential biomarkers. The causal relationships between these biomarkers and OA, as well as their biological functions, were further explored. **Results**: Fourteen candidate genes were identified for machine learning analysis, with *DDIT3*, C*E*BPB, *CX3CR1*, and *ARHGAP25* emerging as feature genes. *CEBPB* and *CX3CR1*, which exhibited AUCs > 0.7 in the GSE55235 and GSE55457 datasets, were selected as potential biomarkers. Notably, *CEBPB* expression was lower, while *CX3CR1* expression was elevated in the case group. Furthermore, both genes were co-enriched in spliceosome, lysosome, and cell adhesion molecule pathways. MR analysis confirmed that *CEBPB* and *CX3CR1* were causally linked to OA and acted as protective factors (IVW model for *CEBPB*: OR = 0.9051, *p* = 0.0001; IVW model for *CX3CR1*: OR = 0.8141, *p* = 0.0282). **Conclusions**: *CEBPB* and *CX3CR1* were identified as potential chemokine-related biomarkers, offering insights into OA and suggesting new avenues for further investigation.

## 1. Introduction

Osteoarthritis (OA), a prevalent chronic joint disorder, is primarily characterized by the degeneration of joint cartilage and inflammation of surrounding tissues [1]. This condition results in joint pain, stiffness, swelling, and functional impairment, significantly reducing patients’ quality of life [2]. OA ranks among the most common joint diseases globally, with a higher prevalence in the elderly population [3]. However, it also affects middle-aged and younger individuals, particularly those with a history of joint injuries or deformities. The incidence of OA is influenced by several factors, including aging, obesity, genetic predisposition, and joint trauma. OA progression is gradual, with symptoms worsening over time, and severe cases may lead to disability [4]. Current treatment approaches primarily focus on symptom management, including pain relief, anti-inflammatory drugs, physical therapy, and joint replacement surgery [5,6,7]. These therapies, however, provide only symptomatic relief and do not prevent disease progression or repair damaged joint cartilage. Consequently, identifying new therapeutic strategies has become a critical focus in OA research. Recently, an increasing number of studies have concentrated on the discovery and application of biomarkers to aid in diagnosing OA, assessing disease progression, and guiding personalized treatment [8,9].

Chemokines are small molecular proteins that play a pivotal role in cell chemotaxis, activation, and the regulation of immune cell migration [10]. In the context of inflammation and immune responses, chemokines are essential for cell regulation and tissue localization [11]. The chemokine family includes several subtypes, such as CC, CXC, C, and CX3C, with CC motif chemokine ligands (CCL) and their corresponding receptors (CCR) being particularly important [12] in disease onset and progression [13,14,15,16]. Abnormal expression of CCL and CCR is frequently associated with exacerbated inflammation and disease progression in various inflammatory conditions [17]. Despite extensive research into the roles of chemokines and related molecules across numerous diseases, their precise mechanisms in joint diseases like OA remain unclear and warrant further investigation [18].

Mendelian randomization (MR) has emerged as a key method for investigating the causal mechanisms of disease [19]. By simulating randomized controlled trials using naturally occurring genetic variations, MR evaluates the causal effects of specific genes on phenotypic outcomes [20]. However, MR studies focused on OA remain limited, and the influence of chemokine-related genes (CRGs) on OA is not yet fully understood [21]. Therefore, further MR studies are essential for a comprehensive understanding of the role of chemokines in OA.

For the first time, this study integrates transcriptomic, GWAS, and eQTL data related to OA, combined with Mendelian randomization analysis and machine learning approaches, to identify potential chemokine-related biomarkers and establish a novel framework for OA research. On this basis, functional, regulatory mechanism, and causal relationship analyses were conducted to provide a theoretical foundation for the diagnostic value of chemokines in OA. This work aims to offer new perspectives and methodologies for OA etiology and treatment, thereby proposing more effective diagnostic and therapeutic strategies to improve the overall management of OA.

## 2. Materials and Methods

### 2.1. Data Sources

The training set (GSE55235) and validation set (GSE55457) were sourced from the GEO database (https://www.ncbi.nlm.nih.gov/gds, accessed on 8 September 2023). These datasets included ten synovial tissue samples from osteoarthritic joints and ten synovial tissue samples from healthy joints (control group), respectively. CRGs, including CCL and CCR, were obtained from prior studies and encompassed inflammatory chemokines, homeostatic chemokines, and bifunctional chemokines [18]. Summary-level data for OA (ebi-a-GCST005810) and potential biomarkers (eqtl-a-ENSG00000172216 and eqtl-a-ENSG00000168329) were downloaded from the IEU OpenGWAS database (https://gwas.mrcieu.ac.uk/, accessed on 8 September 2023). The ebi-a-GCST005810 dataset consisted of 15,543,628 single-nucleotide polymorphisms (SNPs) from 11,989 European samples.

### 2.2. Weighted Gene Coexpression Network Construction Analysis (WGCNA)

The ssGSEA algorithm in the “GSVA” package was used to calculate the CCL and CCR scores for each sample in the GSE55235 dataset to identify module genes associated with CC chemokine ligands and receptors [22]. The “GoodSamplesGenes” function in the “WGCNA” package was then applied to cluster all samples in the training set, and outlier samples were identified and excluded to ensure the accuracy of the analysis [23]. To assess whether genes exhibited similar expression patterns, the scale-free fit index (R^2^) was set to 0.80. Optimal soft thresholding, with values exceeding 0.80 and a mean connectivity near 0, was selected to construct a scale-free co-expression network. Based on this threshold, the minModuleSize was set to 200, and gene modules were obtained using the hybrid dynamic tree cutting algorithm. CCL and CCR scores were introduced as traits, and Pearson correlation analysis was performed between traits and module genes using the “cor” function in the “corrplot” package (|correlation| > 0.3, *p* < 0.05) [24]. A correlation heatmap was generated using the “ggplot2” package. The module with the highest correlation was selected for subsequent analysis.

### 2.3. Differential Expression Analysis

Differentially expressed genes (DEGs) between OA and control samples were identified through differential expression analysis in GSE55235 using the “limma” package (|log_2_FC| > 0.5, adj. *p* < 0.05) [25]. Volcano plots and heatmaps were created to visualize the expression of these DEGs.

### 2.4. Identification of Candidate Genes

To further narrow down candidate genes, DEGs and key module genes were overlapped to identify DE-CRGs. MR analysis was then performed using the “TwoSampleMR” package to select genes with *p*-values < 0.05 for IVW analysis and level effects > 0.05 for subsequent evaluation [26]. Gene Ontology (GO) term and Kyoto Encyclopedia of Genes and Genomes (KEGG) pathway analyses were conducted using the “ClusterProfiler” package to explore the functional roles of these genes (adj. *p* < 0.05) [27]. Genes identified through MR analysis were imported into the STRING database to investigate gene interactions and construct a protein–protein interaction (PPI) network using “Cytoscape 3.8.0 software” [28]. The top 20 genes, ranked by normalized cross-correlation (NCC) and DNMC methods in the cytoHubba plug-in, were intersected to select candidate genes.

### 2.5. Identification of Potential Biomarkers, Establishment of Nomogram, and Expression Validation

To further identify genes closely related to OA, candidate genes in the training set were screened using two machine learning algorithms: LASSO analysis and SVM-RFE. LASSO, with 10-fold cross-validation, was performed using the “glmnet” package [29], and the optimal genes were selected when the lambda value was minimized. SVM-RFE analysis was carried out using the “caret” package [30], and the optimal gene combination was determined by selecting the point with the lowest error rate. The intersection of the LASSO and SVM-RFE genes was obtained using the “ggvenn” package [31] to identify feature genes. The diagnostic value of these feature genes was assessed with the “pROC” package by plotting ROC curves for each gene in the GSE55235 and GSE55457 datasets. Genes with areas under the curve (AUCs) > 0.7 were selected as potential biomarkers. A nomogram model was then constructed based on these biomarkers using the “rms” package [32], and calibration curves were generated to validate the model’s efficacy. The expression levels of the potential biomarkers were further validated in the GSE55235 and GSE55457 datasets, with the Wilcoxon test applied to compare differences between OA and control samples (*p* < 0.05).

### 2.6. Gene Set Enrichment Analysis (GSEA)

To investigate the biological pathways associated with the potential biomarkers, the “c2.cp.kegg.v7.4.symbols.gmt” reference gene set from the MSigDB database (https://www.gsea-msigdb.org/gsea/msigdb, accessed on 10 September 2023) was used. Pearson correlations between the potential biomarkers and other genes in each sample were calculated using the “psych” package [33], with genes sorted by correlation coefficients. GSEA was then performed using the “clusterProfiler” package, with a normalized enrichment score (NES) > 1 and adjusted *p*-value < 0.05. The top 5 pathways with the most significant *p*-values were displayed for analysis [27].

### 2.7. MR Analysis

Exposure factor reading and filtering were conducted using the “extract_instruments” function from the “TwoSampleMR” package with a *p*-value threshold of <5 × 10^−8^ for MR analysis [26]. SNPs for linkage disequilibrium analysis (LDA) were removed (clump = TRUE, r^2^ = 0.001, kb = 10000), and the F-statistic was calculated. SNPs were considered sufficiently robust when F > 10. Instrumental variables (IVs) strongly correlated with exposure factors were then selected for MR analysis. Three key assumptions underpin MR studies: (1) IVs must be strongly correlated with exposure factors, (2) IVs must be independent of other confounding factors, and (3) IVs must influence the outcome only through the exposure factors.

The “Harmonize_data” function from the “TwoSampleMR” package was used to harmonize effect equivalents and effect sizes. The primary MR methods employed were MR–Egger [34], weighted median [35], inverse-variance weighted (IVW) [36], simple mode [26], and weighted mode [37]. Among these, IVW was considered the most crucial method due to its superior ability to detect causal relationships. A risk factor was identified when the odds ratio (OR) exceeded 1, while an OR below 1 indicated a protective factor. Scatter plots, forest plots, and funnel plots were generated to visualize the results. To assess the reliability of the MR analysis, a sensitivity analysis was conducted. First, heterogeneity was tested using Cochran’s Q test (*p* > 0.05). Second, a horizontal pleiotropy test was performed (*p* > 0.05). Finally, the leave-one-out (LOO) method was applied, systematically removing each SNP. If the exclusion of any SNP did not significantly alter the outcome, this indicated the robustness of the MR analysis.

### 2.8. Network Construction

The ChEA3 database (https://maayanlab.cloud/chea3/, accessed on 10 September 2023) was used to predict transcription factor (TF)-targeting potential biomarkers. The starBase database (http://mirdb.org/, accessed on 10 September 2023) was used to identify the miRNAs targeting these potential biomarkers (pancancerNum ≥ 6). The “Cytoscape software” was then used to visualize the miRNA-biomarker-TF network. Furthermore, to explore the interactions between potential biomarkers and drugs, the CTD database (https://ctdbase.org/, accessed on 10 September 2023) was used to predict potential drugs associated with the biomarkers, and a biomarker-drug network was constructed.

### 2.9. Statistical Analysis

R software (v 4.2.3) was employed for data processing and analysis. Group comparisons were performed using the Wilcoxon test, with a *p*-value of < 0.05 considered statistically significant (*p* < 0.05).

## 3. Results

### 3.1. Recognition of DE-CRGs

**Figure 1:**
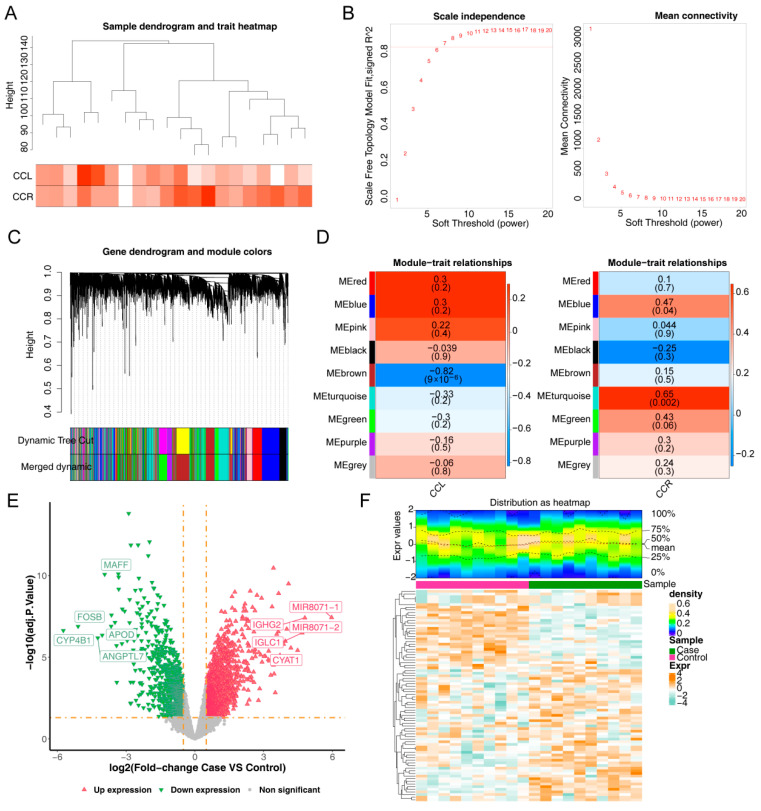
WGCNA and differential expression analysis in the training set. (**A**) Sample clustering tree. (**B**) Identification of the soft threshold (the horizontal axis in both cases represented the weighting parameter, the power value. In the left—hand figure, the vertical axis represented the square of the fitting coefficient between log(k) and log(p(k)) in the corresponding network, namely signedR^2^. The higher the square of the correlation coefficient, the closer the network approximates a scale-free distribution. In the right-hand figure, the vertical axis represented the mean value of the adjacency functions of all genes in the corresponding gene module). (**C**) Cluster dendrogram (different colors represented different modules. By default, genes that cannot be classified into any module were colored in gray). (**D**) Relationships between modules and traits (CCL and CCR) (the vertical axis represented different modules, and the horizontal axis represented clinical traits. Each square denoted the correlation coefficient between a certain module and a certain trait. Blue indicated negative correlation, while red indicated positive correlation). (**E**) Volcano plot of DEGs (the horizontal axis represented the fold change in gene expression, and the vertical axis represented the adj.p.value). (**F**) Heatmap of DEGs (the top graph showed the density distribution of the expression levels of differentially expressed genes. The bottom graph showed the heatmap of the expression levels of differentially expressed genes).

To identify genes associated with the CCL and CCR scores, WGCNA was performed. Clustering analysis revealed no outlier samples, indicating that subsequent analyses could proceed (Figure 1A). The optimal soft threshold was determined to be seven, at which point the interactions among genes best conformed to a scale-free distribution (Figure 1B). Based on this threshold, nine modules were identified (Figure 1C). The MEbrown module, which showed a negative correlation with the CCL score (cor = −0.82), and the MEturquoise module, which exhibited a positive correlation with the CCR score (cor = 0.65), were selected for further analysis (Figure 1D). A total of 6339 genes in these modules were selected as key module genes for subsequent analysis. Differential expression analysis identified 1797 DEGs from the GSE55235 dataset (case vs. control), which included 1084 overexpressed genes and 713 underexpressed genes. A volcano plot and heatmap were generated to visualize the expression of DEGs (Figure 1E,F). By overlapping the DEGs with the key module genes, 1466 DE-CRGs were identified for further study.

### 3.2. CEBPB and CX3CR1 Were Identified as Potential Biomarkers

**Figure 2:**
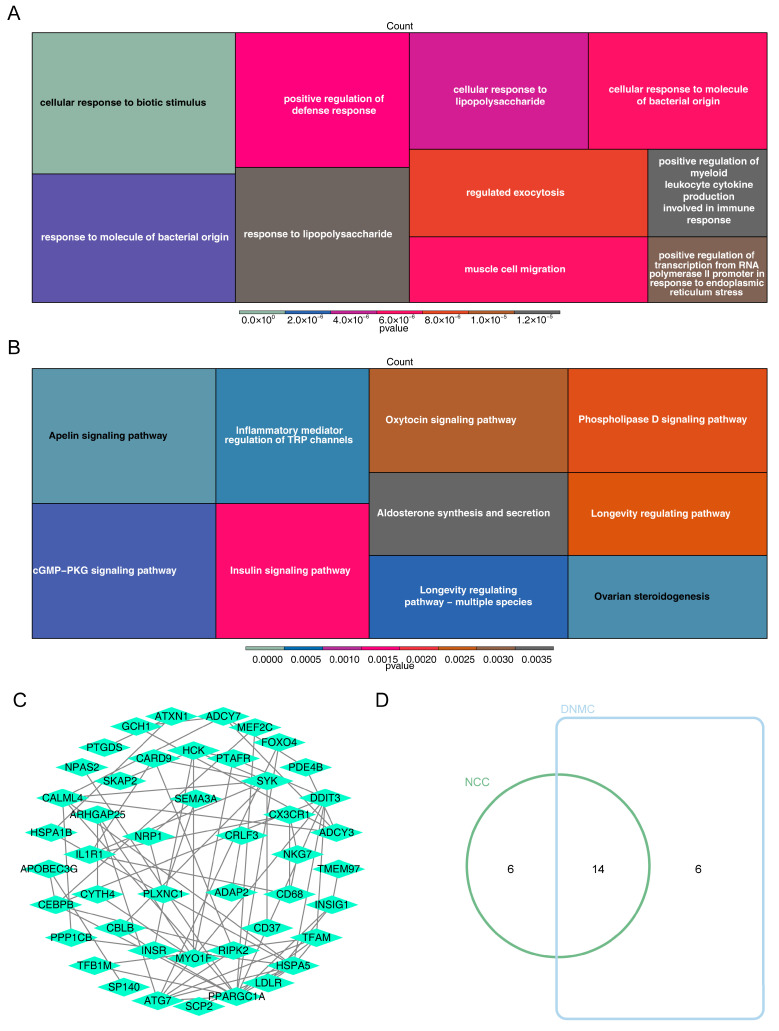
Functional analysis of 82 genes and identification of candidate genes. (**A**) GO enrichment results for 82 genes. (**B**) KEGG enrichment results for 82 genes. (**C**) PPI results of 82 genes. (**D**) Venn diagram of the NCC and DNMC algorithms.

The DE-CRGs were incorporated into the MR analysis, resulting in the identification of 82 genes with a causal relationship to OA (Table 1), offering valuable insights into the disease’s pathogenesis. These genes were primarily involved in the apelin signaling pathway and the cellular response to biotic stimulus pathway (Figure 2A,B), suggesting that further exploration of these pathways could unveil novel therapeutic targets for OA. The PPI network analysis revealed intricate interactions between *CEBPB*, *CX3CR1*, and other genes, including *ANK1* and *CRLF3* (Figure 2C). After intersecting the genes identified through the NCC and DNMC algorithms, 14 candidate genes were ultimately selected for further analysis (Figure 2D).

**Figure 3:**
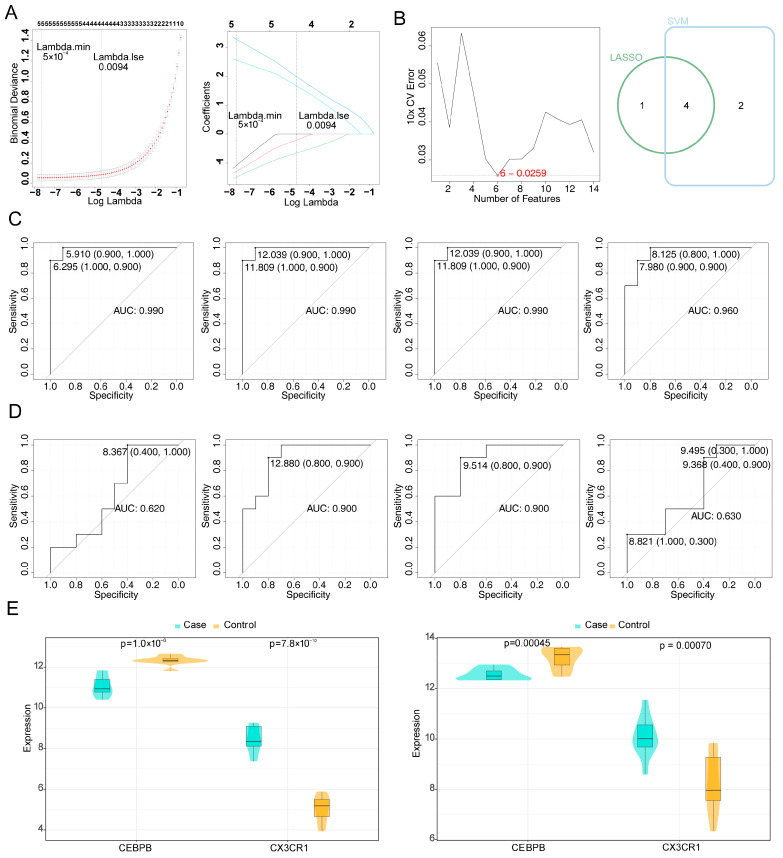
Screening for potential biomarkers. (**A**) Results of LASSO regression analysis for 14 candidate genes (in the left graph, the horizontal axis represents the log(lambda) value, and the vertical axis represents the degree of freedom. In the right graph, the horizontal axis represents the log(lambda), and the vertical axis represents the coefficient of the gene). (**B**) Results of SVM-RFE analysis for 14 candidate genes (the vertical axis is labeled as “10xCV Error”, which represents the ten-fold cross-validation error that was used to evaluate the generalization ability of the model. The curve shows the fluctuation of the ten-fold cross-validation error as the variable on the horizontal axis changed) and Venn diagram of two machine learning algorithms. (**C**,**D**) ROC curves analysis (the vertical axis represents sensitivity, and the horizontal axis represents specificity). (**E**) Results of gene expression analyses in the training and testing sets (the horizontal axis represents potential biomarkers, and the vertical axis represents the expression level).

The LASSO analysis identified five feature genes—*DDIT3*, *TFAM*, *CEBPB*, *CX3CR1*, and *ARHGAP25*—when the lambda value was set to 5 × 10^−4^. Similarly, the SVM-RFE analysis revealed six feature genes—*DDIT3*, *CX3CR1*, *CEBPB*, *PTAFR*, *ARHGAP25*, and *MYO1F*—at the point of lowest error rate. The intersection of the feature genes from both methods resulted in four common genes: *DDIT3*, *CEBPB*, *CX3CR1*, and *ARHGAP25* (Figure 3A,B). ROC curve analysis in both the training and testing sets revealed that *CEBPB* and *CX3CR1* had AUCs greater than 0.7, thus confirming them as potential biomarkers (Figure 3C,D). Gene expression analysis in the GSE55235 and GSE55457 datasets indicated that *CEBPB* was expressed at low levels, while *CX3CR1* was highly expressed in the case group (Figure 3E).

**Figure 4:**
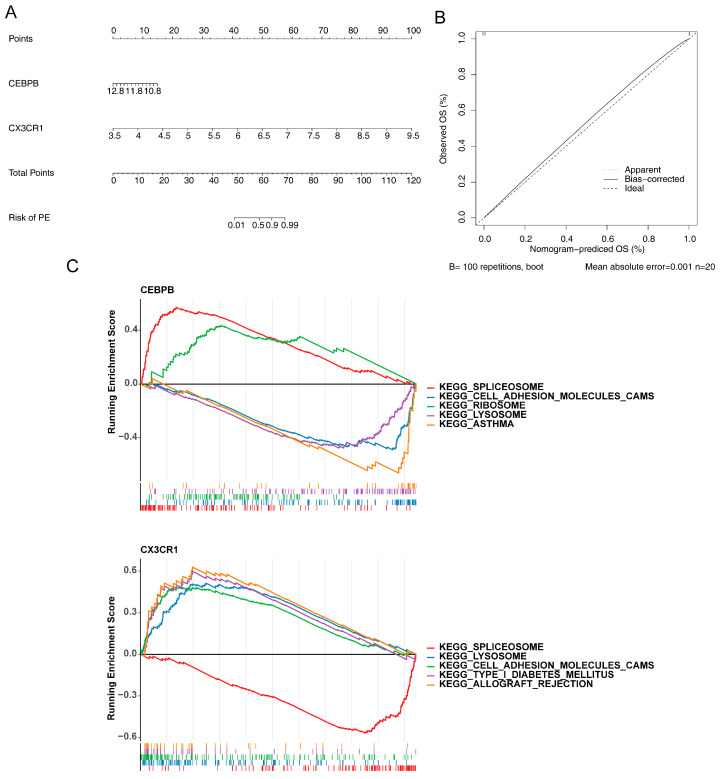
Construction and validation of the nomogram and functional analysis of potential biomarkers. (**A**) Nomogram of potential biomarkers. (**B**) Calibration curve of the nomogram. (**C**) GSEA results for potential biomarkers (CEBPB and CX3CR1).

A nomogram was constructed for *CEBPB* and *CX3CR1*, where each potential biomarker corresponded to a specific point, and the sum of these points represented the total score. This total score could predict the prevalence of OA and was positively correlated with its incidence (Figure 4A). Calibration curve analysis showed that the curve closely approximated 1, indicating the high diagnostic accuracy of the nomogram (Figure 4B). This tool offers a valuable resource for facilitating communication between doctors and patients, enabling physicians to use the nomogram to select tailored, personalized treatment plans. Additionally, GSEA was performed to explore the biological functions of the biomarkers in greater detail. The results demonstrated that *CEBPB* and *CX3CR1* were co-enriched in spliceosome, lysosome, and cell-adhesion molecule (CAM) pathways (Figure 4C). Abnormal spliceosome function could disrupt gene expression accuracy, leading to dysfunctions in related cells such as chondrocytes. Changes in lysosomal function may impair the degradation and metabolism of cellular substances, further affecting cartilage tissue homeostasis. Alterations in CAMs could disrupt cell–cell interactions and adhesion between cells and the extracellular matrix, thereby influencing the structure and function of articular cartilage. The co-enrichment of CEBPB and CX3CR1 in these pathways suggests that they may contribute to OA pathogenesis through a synergistic effect.

### 3.3. CEBPB and CX3CR1 Were Causally Associated with OA

**Figure 5:**
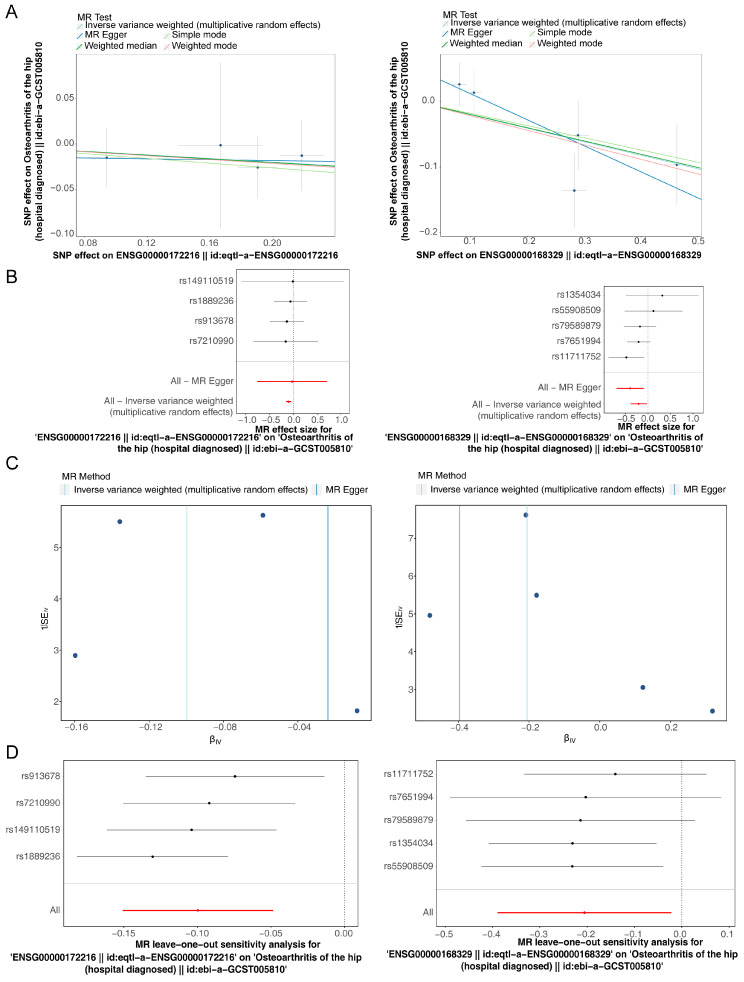
MR results. (**A**) Scatter plots of SNPs associated with two potential biomarkers and OA (the lines in the figure represented five algorithms). (**B**) Forest plot results for the two potential biomarkers. (**C**) Funnel plot results for the two potential biomarkers (the horizontal axis is βiv, which represents the effect estimate calculated by the inverse-variance weighted method. The vertical axis was 1/SEiv, where SEiv represented the standard error. The 1/SEiv could reflect the precision of the effect estimate, and a larger value indicates higher precision). (**D**) Leave-one-out results for two potential biomarkers (the horizontal axis represents the estimated effect, and the vertical axis represents different single-nucleotide polymorphism (SNP) loci).

After IV screening, a total of four SNPs related to *CEBPB* and five SNPs related to *CX3CR1* were identified (Table S1). The MR analysis demonstrated that *CEBPB* and *CX3CR1* were causally associated with OA and identified as protective factors (IVW model for *CEBPB*: OR = 0.9051, *p* = 0.0001; IVW model for *CX3CR1*: OR = 0.8141, *p* = 0.0282) (Table 2 and Table 3). The negative slope observed in the IVW method’s scatter plot suggested that higher levels of *CEBPB* and *CX3CR1* were associated with a reduced risk of developing OA (Figure 5A). This relationship was further validated by the forest plot (Figure 5B). Additionally, the SNPs displayed a roughly symmetrical distribution on both sides of the plot, supporting the alignment with the second law of Mendelian inheritance (Figure 5C). Sensitivity analysis indicated no heterogeneity, as confirmed by the Cochran’s Q test (*p* > 0.05) (Table 4). The horizontal pleiotropy test also revealed no pleiotropic effects in the MR analysis (*p* > 0.05) (Table 5). Furthermore, LOO analysis showed no significant bias, reinforcing the reliability of the overall findings (Figure 5D).

### 3.4. Complex Interactions Between Potential Biomarkers

**Figure 6:**
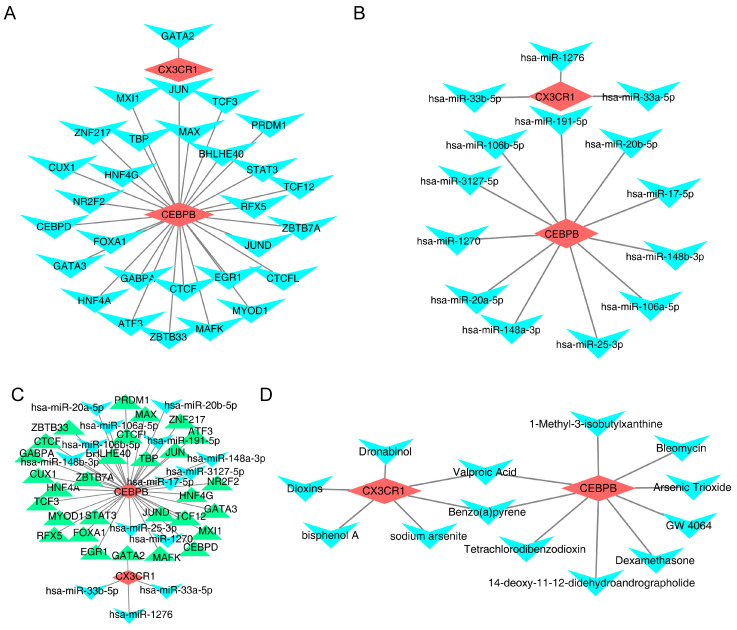
TF, miRNA, and drug prediction. (**A**): CEBPB and CX3CR1 target transcription factors (red color represents potential biomarkers, and blue color represents TFs), (**B**): CEBPB and CX3CR1 target miRNAs (red color represents potential biomarkers, and blue color represents miRNAs), (**C**): visualization of miRNA-biomarker-TF networks (red color represents potential biomarkers, blue color represents miRNAs, and green color represents TFs), (**D**): CTD constructing biomarker–drug networks (red color represents potential biomarkers, and blue color represents drugs).

To explore the regulatory mechanisms of the potential biomarkers, target TFs and miRNAs were predicted via a database, identifying 29 TFs and 11 miRNAs for *CEBPB*, and one TF and three miRNAs for *CX3CR1* (Figure 6A,B). A regulatory network was constructed, illustrating interactions such as GATA2-*CX3CR1*-hsa-miR-1276 (Figure 6C). This highlights the complex roles these components play in gene expression regulation, providing insights into the molecular processes involved in the onset and progression of OA. Furthermore, a regulatory relationship was identified between valproic acid and benzo(a)pyrene, both of which are potential biomarkers with therapeutic implications for OA (Figure 6D).

## 4. Discussion

Recent research has firmly established that persistent, low-intensity inflammation, encompassing both innate and adaptive immune responses, significantly influences the onset and progression of OA [38,39]. The interplay between CCLs and CCRs leads to the recruitment of various immune cells into the injured joints, contributing to local inflammation [40,41]. Additionally, within the nerve endings of the knee joint, CCLs, CCRs, and cytokines initiate the release of spinal neurotransmitters, causing hyperalgesia [42,43]. Consequently, this study identified two potential biomarkers, CEBPB and CX3CR1, using bioinformatics methods. These biomarkers can serve as quantitative indicators to predict disease progression, joint function deterioration, and patient response to treatment more accurately, facilitating the development of more proactive intervention strategies. Moreover, although various treatment options exist for late-stage OA, including pharmacotherapy, physical therapy, and surgical interventions, patients’ responses can vary. Identifying potential biomarkers can help uncover individual biological characteristics, enabling the customization of the most effective treatment plans. These biomarkers can also aid in monitoring disease recurrence, assisting physicians in promptly assessing changes in the conditions of patients with late-stage OA, adjusting treatment plans accordingly, and preventing further disease progression.

CEBPB, or CCAAT/enhancer-binding protein beta, is a TF in the C/EBP family. It can be activated by various inflammatory stimuli such as IL-17 and LPS, subsequently modulating multiple genes involved in the inflammatory process [44]. The upregulation of CEBPB in Alzheimer’s disease promotes the expression of proinflammatory genes in microglia and affects macrophage activation [44]. CEBPB also plays a role in dendritic cells and in autoimmune disorders of the central nervous system [45]. In patients with amyotrophic lateral sclerosis (ALS), CEBPB expression was elevated in lymphocytes and nerve tissue, making it a potential marker for ALS progression [46,47]. This suggests a strong connection between CEBPB and inflammatory processes in nerve tissues, indicating its involvement in various neurological inflammatory responses. Autoimmunity and inflammation are closely linked to OA’s development and progression. Furthermore, CEBPB is associated with macrophage-related pathways [48], suggesting a correlation with OA’s pathological process. Notably, CEBPB is a gene connected to both OA and metabolic syndrome, and it holds diagnostic value for OA individuals with metabolic syndrome [49]. Wang et al. found that 5,7,3’,4’-tetramethoxyflavone inhibits extracellular matrix degradation in OA by modulating the C/EBPβ/ADAMTS5 signaling pathway [50]. Nevertheless, its precise function in inflammation-associated disorders is still a matter of debate and warrants more in-depth studies [51]. In the present study, MR analysis revealed that CEBPB serves as a therapeutic target for OA, showing a causal relationship with the disease. MR effectively minimizes confounding factors and reverse causation, identifying CEBPB as a protective factor for OA. This provides stronger evidence for the causal link, offering a deeper and more comprehensive understanding of the relationship between CEBPB and OA.

CX3CR1, or CX3C chemokine receptor 1, is a G protein-coupled receptor that primarily interacts with the chemokine CX3CL1 (also known as fractalkine or neurotactin) [52]. While an expression correlation analysis of clinical samples has identified CX3CL1 as a potential biomarker for knee osteoarthritis, its receptor CX3CR1 remains unreported in this regard, suggesting a gap that merits further investigation [53]. It is found on the inner lining of synovial fibroblasts in the knee joint, where CX3CR1-positive macrophages form a dense physical barrier with CX3CR1, isolating the joint space from the external environment and protecting the joint [54]. These findings suggest that CX3CR1 may influence the pathogenesis of OA. Notably, the MR analysis in this study revealed a causal relationship between CX3CR1 and OA, with CX3CR1 acting as a protective factor against the disease. This result is consistent with most previous studies, providing a solid theoretical basis for the clinical diagnosis, treatment, and prognosis of OA.

Through database searches, this study predicted the target TFs and miRNAs for the potential biomarkers. Previous research suggests that the target miRNA of CX3CR1, hsa-miR-1276, may be linked to the development of cardiovascular diseases [55,56]. Hsa-miR-33a-5p is potentially associated with chemoresistance in hepatocellular carcinoma [57], while hsa-miR-33b-5p may be related to type 2 diabetes, myocardial infarction, and other conditions [58,59]. Notably, the relationships between these target miRNAs of CX3CR1 and OA have not been explored. Our study is the first to propose that these three miRNAs could be involved in OA development via CX3CR1.

The database search identified 11 target miRNAs for CEBPB. Previous studies have suggested that hsa-miR-20b-5p is implicated in diseases such as atrial fibrillation and liver cirrhosis [60,61], while hsa-miR-106b-5p is linked to pulmonary hypertension and melanoma progression [62,63]. These 11 target miRNAs of CEBPB have not been previously associated with OA development. Our study is the first to propose a potential connection, offering new insights into the mechanisms underlying OA.

This study is the first to systematically construct a miRNA–mRNA regulatory network centered on CX3CR1 and CEBPB, identifying several miRNAs previously unreported in the context of osteoarthritis (OA). This provides a novel perspective for exploring post-transcriptional regulatory mechanisms in OA. For example, hsa-miR-1276, a predicted regulator of CX3CR1, has been associated with the pathogenesis of cardiovascular diseases [55,56]. hsa-miR-33a-5p may be involved in chemotherapy resistance in hepatocellular carcinoma [57], while hsa-miR-33b-5p plays significant roles in type 2 diabetes and myocardial infarction [58,59]. In addition, among the miRNAs targeting CEBPB, hsa-miR-20b-5p has been implicated in atrial fibrillation and liver cirrhosis [60,61], and hsa-miR-106b-5p is known to contribute to the progression of pulmonary arterial hypertension and melanoma [62,63]. Although these miRNAs have been demonstrated to exert important biological functions in various diseases, their relevance to OA has not yet been established. The findings of this study offer new directions and molecular clues for future mechanistic investigations in OA.

Additionally, 29 TFs that target CEBPB were predicted in this study. Several of these TFs have been confirmed to play roles in the development and progression of OA. For example, JUND promotes OA progression via the miR-423-5p/KDM5C axis and induces immune inflammation [64,65]. ATF3 has been identified as a potential diagnostic marker for OA and is involved in synovial immunity and chondrocyte death [66,67,68,69]. Upregulation of TCF12 is known to lead to OA progression [70], and GATA3 is associated with cartilage damage during OA development [71,72]. Both TBP and MXI1 are linked to the occurrence and progression of OA [73,74]. Previous studies have connected GATA2 to rheumatism [75], and PRDM1 may be associated with Alzheimer’s disease development [76]. However, other target TFs of CEBPB have not been previously discussed in relation to OA progression, suggesting new avenues for research into their involvement in OA.

Both valproic acid and benzo[a]pyrene were found to have regulatory relationships with two potential biomarkers, suggesting their therapeutic potential for OA. Valproic acid, an anticonvulsant and mood stabilizer, operates through multiple mechanisms and is mainly used to treat epilepsy and bipolar disorder. Previous studies have demonstrated that VPA can influence neurotransmitter levels and regulate gene expression, though the precise mechanisms, particularly regarding specific pathways and targets in various disease states, remain unclear [77]. Further research could shed light on these mechanisms. Benzo[a]pyrene, primarily studied for its carcinogenic effects, has been shown to induce DNA double-strand breaks [78]. Future research could explore whether its therapeutic effects on OA involve gene regulation in synovial cells.

This study identified two potential biomarkers, CEBPB and CX3CR1, through bioinformatics analysis. MR analysis revealed a significant causal relationship between these biomarkers and OA, establishing that both genes serve as protective factors against OA. Based on these findings, a series of in-depth analyses were conducted to explore the functions and potential regulatory mechanisms of these genes. Although bioinformatics analysis has provided significant insights and direction for our research, the certainty and broad applicability of these results are somewhat limited due to the lack of validation through biological experiments. We are fully aware of the indispensable nature of experimental validation in biology. The reason for not conducting related experiments in this study mainly stems from resource limitations, time constraints, and difficulties in sample collection. Despite these challenges, we are firmly committed to conducting experimental validations and have developed a detailed future work plan. We will verify our findings through animal and cell experiments in the future, providing more effective strategies and methods for the diagnosis and treatment of OA. Additionally, the current data and types may not fully support all conclusions related to the early detection of OA biomarkers. To address this limitation, future research will focus on collaboration with other research teams or medical institutions, enabling access to broader and more representative patient data through data sharing or joint research initiatives. This collaboration will enhance the database and deepen the investigation into early biomarkers of OA.

## Figures and Tables

**Table 1:** Mendelian randomization analysis of DE-CRGs.

Gene	id.Exposure	id.Outcome	Outcome	Exposure	Method	nsnp	b	se	pval	p_no	Level Test
SKAP2	eqtl-a-ENSG00000005020	ebi-a-GCST005810	Osteoarthritis of the hip (hospital-diagnosed) || id:ebi-a-GCST005810	ENSG00000005020 || id:eqtl-a-ENSG00000005020	Inverse-variance weighted (multiplicative random effects)	12	−0.06355	0.029	0.028	MR Egger weighted median	0.964302
ANK1	eqtl-a-ENSG00000029534	ebi-a-GCST005810	Osteoarthritis of the hip (hospital-diagnosed) || id:ebi-a-GCST005810	ENSG00000029534 || id:eqtl-a-ENSG00000029534	Inverse-variance weighted (multiplicative random effects)	4	−0.28875	0.06493	0	MR Egger	0.604415
TFB1M	eqtl-a-ENSG00000029639	ebi-a-GCST005810	Osteoarthritis of the hip (hospital-diagnosed) || id:ebi-a-GCST005810	ENSG00000029639 || id:eqtl-a-ENSG00000029639	Inverse-variance weighted (multiplicative random effects)	3	−0.22194	0.051988	0	MR Egger	0.787497
HSPA5	eqtl-a-ENSG00000044574	ebi-a-GCST005810	Osteoarthritis of the hip (hospital-diagnosed) || id:ebi-a-GCST005810	ENSG00000044574 || id:eqtl-a-ENSG00000044574	Inverse-variance weighted (multiplicative random effects)	5	0.156907	0.04502	0	MR Egger weighted median	0.953288
ASB1	eqtl-a-ENSG00000065802	ebi-a-GCST005810	Osteoarthritis of the hip (hospital-diagnosed) || id:ebi-a-GCST005810	ENSG00000065802 || id:eqtl-a-ENSG00000065802	Inverse-variance weighted (multiplicative random effects)	3	0.095562	0.048124	0.047	MR Egger weighted median	0.476947
SEMA3A	eqtl-a-ENSG00000075213	ebi-a-GCST005810	Osteoarthritis of the hip (hospital-diagnosed) || id:ebi-a-GCST005810	ENSG00000075213 || id:eqtl-a-ENSG00000075213	Inverse-variance weighted (multiplicative random effects)	3	−0.27709	0.017105	0	MR Egger	0.986775
SP140	eqtl-a-ENSG00000079263	ebi-a-GCST005810	Osteoarthritis of the hip (hospital-diagnosed) || id:ebi-a-GCST005810	ENSG00000079263 || id:eqtl-a-ENSG00000079263	Inverse-variance weighted (multiplicative random effects)	3	−0.17196	0.086501	0.047	MR Egger	0.840832
EPB41L2	eqtl-a-ENSG00000079819	ebi-a-GCST005810	Osteoarthritis of the hip (hospital-diagnosed) || id:ebi-a-GCST005810	ENSG00000079819 || id:eqtl-a-ENSG00000079819	Inverse-variance weighted (multiplicative random effects)	3	0.121946	0.046439	0.009	MR Egger weighted median	0.62119
MEF2C	eqtl-a-ENSG00000081189	ebi-a-GCST005810	Osteoarthritis of the hip (hospital-diagnosed) || id:ebi-a-GCST005810	ENSG00000081189 || id:eqtl-a-ENSG00000081189	Inverse-variance weighted (multiplicative random effects)	5	−0.13635	0.069189	0.049	MR Egger weighted median	0.38673
OVGP1	eqtl-a-ENSG00000085465	ebi-a-GCST005810	Osteoarthritis of the hip (hospital-diagnosed) || id:ebi-a-GCST005810	ENSG00000085465 || id:eqtl-a-ENSG00000085465	Inverse-variance weighted (multiplicative random effects)	3	−0.09836	0.03839	0.01	MR Egger weighted median	0.725835
NRP1	eqtl-a-ENSG00000099250	ebi-a-GCST005810	Osteoarthritis of the hip (hospital-diagnosed) || id:ebi-a-GCST005810	ENSG00000099250 || id:eqtl-a-ENSG00000099250	Inverse-variance weighted (multiplicative random effects)	6	−0.12929	0.037849	0.001	MR Egger weighted median	0.866044
CYTH4	eqtl-a-ENSG00000100055	ebi-a-GCST005810	Osteoarthritis of the hip (hospital-diagnosed) || id:ebi-a-GCST005810	ENSG00000100055 || id:eqtl-a-ENSG00000100055	Inverse-variance weighted (multiplicative random effects)	5	−0.17078	0.079644	0.032	MR Egger weighted median	0.716136
SYNGR1	eqtl-a-ENSG00000100321	ebi-a-GCST005810	Osteoarthritis of the hip (hospital-diagnosed) || id:ebi-a-GCST005810	ENSG00000100321 || id:eqtl-a-ENSG00000100321	Inverse-variance weighted (multiplicative random effects)	5	−0.06284	0.029177	0.031	MR Egger weighted median	0.531044
KIAA0930	eqtl-a-ENSG00000100364	ebi-a-GCST005810	Osteoarthritis of the hip (hospital-diagnosed) || id:ebi-a-GCST005810	ENSG00000100364 || id:eqtl-a-ENSG00000100364	Inverse-variance weighted (multiplicative random effects)	4	−0.14508	0.027017	0	MR Egger weighted median	0.75393
HCK	eqtl-a-ENSG00000101336	ebi-a-GCST005810	Osteoarthritis of the hip (hospital-diagnosed) || id:ebi-a-GCST005810	ENSG00000101336 || id:eqtl-a-ENSG00000101336	Inverse-variance weighted (multiplicative random effects)	3	−0.07236	0.013991	0	MR Egger weighted median	0.902966
FNDC3A	eqtl-a-ENSG00000102531	ebi-a-GCST005810	Osteoarthritis of the hip (hospital-diagnosed) || id:ebi-a-GCST005810	ENSG00000102531 || id:eqtl-a-ENSG00000102531	Inverse-variance weighted (multiplicative random effects)	4	−0.09655	0.029565	0.001	MR Egger weighted median	0.745019
NOMO3	eqtl-a-ENSG00000103226	ebi-a-GCST005810	Osteoarthritis of the hip (hospital-diagnosed) || id:ebi-a-GCST005810	ENSG00000103226 || id:eqtl-a-ENSG00000103226	Inverse-variance weighted (multiplicative random effects)	3	−0.11221	0.029073	0	MR Egger weighted median	0.709799
RIPK2	eqtl-a-ENSG00000104312	ebi-a-GCST005810	Osteoarthritis of the hip (hospital-diagnosed) || id:ebi-a-GCST005810	ENSG00000104312 || id:eqtl-a-ENSG00000104312	Inverse-variance weighted (multiplicative random effects)	3	−0.14312	0.034984	0	MR Egger weighted median	0.825168
CD37	eqtl-a-ENSG00000104894	ebi-a-GCST005810	Osteoarthritis of the hip (hospital-diagnosed) || id:ebi-a-GCST005810	ENSG00000104894 || id:eqtl-a-ENSG00000104894	Inverse-variance weighted (multiplicative random effects)	4	−0.12521	0.048795	0.01	MR Egger weighted median	0.440039
NKG7	eqtl-a-ENSG00000105374	ebi-a-GCST005810	Osteoarthritis of the hip (hospital-diagnosed) || id:ebi-a-GCST005810	ENSG00000105374 || id:eqtl-a-ENSG00000105374	Inverse-variance weighted (multiplicative random effects)	3	0.179132	0.057999	0.002	MR Egger weighted median	0.874258
ZKSCAN1	eqtl-a-ENSG00000106261	ebi-a-GCST005810	Osteoarthritis of the hip (hospital-diagnosed) || id:ebi-a-GCST005810	ENSG00000106261 || id:eqtl-a-ENSG00000106261	Inverse-variance weighted (multiplicative random effects)	3	−0.04586	0.009649	0	MR Egger weighted median	0.916197
TNFSF8	eqtl-a-ENSG00000106952	ebi-a-GCST005810	Osteoarthritis of the hip (hospital-diagnosed) || id:ebi-a-GCST005810	ENSG00000106952 || id:eqtl-a-ENSG00000106952	Inverse-variance weighted (multiplicative random effects)	3	−0.1412	0.037591	0	MR Egger weighted median	0.70572
PTGDS	eqtl-a-ENSG00000107317	ebi-a-GCST005810	Osteoarthritis of the hip (hospital-diagnosed) || id:ebi-a-GCST005810	ENSG00000107317 || id:eqtl-a-ENSG00000107317	Inverse-variance weighted (multiplicative random effects)	3	0.234073	0.059862	0	MR Egger	0.564796
TFAM	eqtl-a-ENSG00000108064	ebi-a-GCST005810	Osteoarthritis of the hip (hospital-diagnosed) || id:ebi-a-GCST005810	ENSG00000108064 || id:eqtl-a-ENSG00000108064	Inverse-variance weighted (multiplicative random effects)	7	0.066743	0.029934	0.026	MR Egger weighted median	0.473236
TMEM97	eqtl-a-ENSG00000109084	ebi-a-GCST005810	Osteoarthritis of the hip (hospital-diagnosed) || id:ebi-a-GCST005810	ENSG00000109084 || id:eqtl-a-ENSG00000109084	Inverse-variance weighted (multiplicative random effects)	6	−0.06877	0.026808	0.01	MR Egger weighted median	0.829951
PPARGC1A	eqtl-a-ENSG00000109819	ebi-a-GCST005810	Osteoarthritis of the hip (hospital-diagnosed) || id:ebi-a-GCST005810	ENSG00000109819 || id:eqtl-a-ENSG00000109819	Inverse-variance weighted (multiplicative random effects)	3	0.042277	0.006439	0	MR Egger weighted median	0.929293
PANX1	eqtl-a-ENSG00000110218	ebi-a-GCST005810	Osteoarthritis of the hip (hospital-diagnosed) || id:ebi-a-GCST005810	ENSG00000110218 || id:eqtl-a-ENSG00000110218	Inverse-variance weighted (multiplicative random effects)	5	−0.08089	0.035894	0.024	MR Egger weighted median	0.501225
SLC22A18	eqtl-a-ENSG00000110628	ebi-a-GCST005810	Osteoarthritis of the hip (hospital-diagnosed) || id:ebi-a-GCST005810	ENSG00000110628 || id:eqtl-a-ENSG00000110628	Inverse-variance weighted (multiplicative random effects)	3	0.040591	0.020205	0.045	MR Egger weighted median	0.787391
CBLB	eqtl-a-ENSG00000114423	ebi-a-GCST005810	Osteoarthritis of the hip (hospital-diagnosed) || id:ebi-a-GCST005810	ENSG00000114423 || id:eqtl-a-ENSG00000114423	Inverse-variance weighted (multiplicative random effects)	4	0.062925	0.028786	0.029	MR Egger weighted median	0.754687
TP53I3	eqtl-a-ENSG00000115129	ebi-a-GCST005810	Osteoarthritis of the hip (hospital-diagnosed) || id:ebi-a-GCST005810	ENSG00000115129 || id:eqtl-a-ENSG00000115129	Inverse-variance weighted (multiplicative random effects)	4	0.052781	0.01687	0.002	MR Egger weighted median	0.79608
IL1R1	eqtl-a-ENSG00000115594	ebi-a-GCST005810	Osteoarthritis of the hip (hospital-diagnosed) || id:ebi-a-GCST005810	ENSG00000115594 || id:eqtl-a-ENSG00000115594	Inverse-variance weighted (multiplicative random effects)	3	−0.13532	0.026544	0	MR Egger weighted median	0.906106
SCP2	eqtl-a-ENSG00000116171	ebi-a-GCST005810	Osteoarthritis of the hip (hospital-diagnosed) || id:ebi-a-GCST005810	ENSG00000116171 || id:eqtl-a-ENSG00000116171	Inverse-variance weighted (multiplicative random effects)	5	0.084015	0.036215	0.02	MR Egger weighted median	0.53642
ITGB1BP1	eqtl-a-ENSG00000119185	ebi-a-GCST005810	Osteoarthritis of the hip (hospital-diagnosed) || id:ebi-a-GCST005810	ENSG00000119185 || id:eqtl-a-ENSG00000119185	Inverse-variance weighted (multiplicative random effects)	4	−0.12499	0.061858	0.043	MR Egger weighted median	0.697252
ADCY7	eqtl-a-ENSG00000121281	ebi-a-GCST005810	Osteoarthritis of the hip (hospital-diagnosed) || id:ebi-a-GCST005810	ENSG00000121281 || id:eqtl-a-ENSG00000121281	Inverse-variance weighted (multiplicative random effects)	3	0.080449	0.00958	0	MR Egger weighted median	0.90424
NQO2	eqtl-a-ENSG00000124588	ebi-a-GCST005810	Osteoarthritis of the hip (hospital-diagnosed) || id:ebi-a-GCST005810	ENSG00000124588 || id:eqtl-a-ENSG00000124588	Inverse-variance weighted (multiplicative random effects)	6	0.103165	0.036288	0.004	MR Egger	0.421145
ATXN1	eqtl-a-ENSG00000124788	ebi-a-GCST005810	Osteoarthritis of the hip (hospital-diagnosed) || id:ebi-a-GCST005810	ENSG00000124788 || id:eqtl-a-ENSG00000124788	Inverse-variance weighted (multiplicative random effects)	3	0.083781	0.038075	0.028	MR Egger weighted median	0.697622
DOCK4	eqtl-a-ENSG00000128512	ebi-a-GCST005810	Osteoarthritis of the hip (hospital-diagnosed) || id:ebi-a-GCST005810	ENSG00000128512 || id:eqtl-a-ENSG00000128512	Inverse-variance weighted (multiplicative random effects)	5	−0.37826	0.147865	0.011	MR Egger weighted median	0.638297
KLHDC10	eqtl-a-ENSG00000128607	ebi-a-GCST005810	Osteoarthritis of the hip (hospital-diagnosed) || id:ebi-a-GCST005810	ENSG00000128607 || id:eqtl-a-ENSG00000128607	Inverse-variance weighted (multiplicative random effects)	3	−0.07208	0.035492	0.042	MR Egger weighted median	0.607087
CALML4	eqtl-a-ENSG00000129007	ebi-a-GCST005810	Osteoarthritis of the hip (hospital-diagnosed) || id:ebi-a-GCST005810	ENSG00000129007 || id:eqtl-a-ENSG00000129007	Inverse-variance weighted (multiplicative random effects)	4	−0.02755	0.010673	0.01	MR Egger weighted median	0.837662
CD68	eqtl-a-ENSG00000129226	ebi-a-GCST005810	Osteoarthritis of the hip (hospital-diagnosed) || id:ebi-a-GCST005810	ENSG00000129226 || id:eqtl-a-ENSG00000129226	Inverse-variance weighted (multiplicative random effects)	3	−0.26171	0.015244	0	MR Egger	0.893517
CDO1	eqtl-a-ENSG00000129596	ebi-a-GCST005810	Osteoarthritis of the hip (hospital-diagnosed) || id:ebi-a-GCST005810	ENSG00000129596 || id:eqtl-a-ENSG00000129596	Inverse-variance weighted (multiplicative random effects)	5	0.072609	0.006502	0	MR Egger weighted median	0.927536
LDLR	eqtl-a-ENSG00000130164	ebi-a-GCST005810	Osteoarthritis of the hip (hospital-diagnosed) || id:ebi-a-GCST005810	ENSG00000130164 || id:eqtl-a-ENSG00000130164	Inverse-variance weighted (multiplicative random effects)	8	0.20067	0.04522	0	MR Egger weighted median	0.682704
GCH1	eqtl-a-ENSG00000131979	ebi-a-GCST005810	Osteoarthritis of the hip (hospital-diagnosed) || id:ebi-a-GCST005810	ENSG00000131979 || id:eqtl-a-ENSG00000131979	Inverse-variance weighted (multiplicative random effects)	3	−0.10284	0.032484	0.002	MR Egger weighted median	0.68823
EGLN1	eqtl-a-ENSG00000135766	ebi-a-GCST005810	Osteoarthritis of the hip (hospital-diagnosed) || id:ebi-a-GCST005810	ENSG00000135766 || id:eqtl-a-ENSG00000135766	Inverse-variance weighted (multiplicative random effects)	3	−0.1614	0.065631	0.014	MR Egger weighted median	0.562518
PLXNC1	eqtl-a-ENSG00000136040	ebi-a-GCST005810	Osteoarthritis of the hip (hospital-diagnosed) || id:ebi-a-GCST005810	ENSG00000136040 || id:eqtl-a-ENSG00000136040	Inverse-variance weighted (multiplicative random effects)	3	−0.17868	0.072646	0.014	MR Egger weighted median	0.727798
C7orf25	eqtl-a-ENSG00000136197	ebi-a-GCST005810	Osteoarthritis of the hip (hospital-diagnosed) || id:ebi-a-GCST005810	ENSG00000136197 || id:eqtl-a-ENSG00000136197	Inverse-variance weighted (multiplicative random effects)	8	−0.1108	0.044746	0.013	MR Egger weighted median	0.44153
IFI44	eqtl-a-ENSG00000137965	ebi-a-GCST005810	Osteoarthritis of the hip (hospital-diagnosed) || id:ebi-a-GCST005810	ENSG00000137965 || id:eqtl-a-ENSG00000137965	Inverse-variance weighted (multiplicative random effects)	7	0.179405	0.050254	0	MR Egger weighted median	0.827067
ADCY3	eqtl-a-ENSG00000138031	ebi-a-GCST005810	Osteoarthritis of the hip (hospital-diagnosed) || id:ebi-a-GCST005810	ENSG00000138031 || id:eqtl-a-ENSG00000138031	Inverse-variance weighted (multiplicative random effects)	5	−0.21249	0.101604	0.036	MR Egger weighted median	0.586261
RAB15	eqtl-a-ENSG00000139998	ebi-a-GCST005810	Osteoarthritis of the hip (hospital-diagnosed) || id:ebi-a-GCST005810	ENSG00000139998 || id:eqtl-a-ENSG00000139998	Inverse-variance weighted (multiplicative random effects)	4	−0.25442	0.080683	0.002	MR Egger	0.948296
MFGE8	eqtl-a-ENSG00000140545	ebi-a-GCST005810	Osteoarthritis of the hip (hospital-diagnosed) || id:ebi-a-GCST005810	ENSG00000140545 || id:eqtl-a-ENSG00000140545	Inverse-variance weighted (multiplicative random effects)	4	0.093175	0.044274	0.035	MR Egger weighted median	0.800747
MYO1F	eqtl-a-ENSG00000142347	ebi-a-GCST005810	Osteoarthritis of the hip (hospital-diagnosed) || id:ebi-a-GCST005810	ENSG00000142347 || id:eqtl-a-ENSG00000142347	Inverse-variance weighted (multiplicative random effects)	3	0.350313	0.089257	0	MR Egger weighted median	0.64159
HNMT	eqtl-a-ENSG00000150540	ebi-a-GCST005810	Osteoarthritis of the hip (hospital-diagnosed) || id:ebi-a-GCST005810	ENSG00000150540 || id:eqtl-a-ENSG00000150540	Inverse-variance weighted (multiplicative random effects)	4	−0.07073	0.00656	0	MR Egger weighted median	0.979268
ING1	eqtl-a-ENSG00000153487	ebi-a-GCST005810	Osteoarthritis of the hip (hospital-diagnosed) || id:ebi-a-GCST005810	ENSG00000153487 || id:eqtl-a-ENSG00000153487	Inverse-variance weighted (multiplicative random effects)	3	−0.02985	0.008805	0.001	MR Egger weighted median	0.96251
ARHGAP25	eqtl-a-ENSG00000163219	ebi-a-GCST005810	Osteoarthritis of the hip (hospital-diagnosed) || id:ebi-a-GCST005810	ENSG00000163219 || id:eqtl-a-ENSG00000163219	Inverse-variance weighted (multiplicative random effects)	3	0.162316	0.026159	0	MR Egger weighted median	0.823096
TGFBR2	eqtl-a-ENSG00000163513	ebi-a-GCST005810	Osteoarthritis of the hip (hospital-diagnosed) || id:ebi-a-GCST005810	ENSG00000163513 || id:eqtl-a-ENSG00000163513	Inverse-variance weighted (multiplicative random effects)	4	−0.15754	0.070785	0.026	MR Egger	0.477357
CITED2	eqtl-a-ENSG00000164442	ebi-a-GCST005810	Osteoarthritis of the hip (hospital-diagnosed) || id:ebi-a-GCST005810	ENSG00000164442 || id:eqtl-a-ENSG00000164442	Inverse-variance weighted (multiplicative random effects)	3	0.152919	0.069401	0.028	MR Egger weighted median	0.57333
GALNT10	eqtl-a-ENSG00000164574	ebi-a-GCST005810	Osteoarthritis of the hip (hospital-diagnosed) || id:ebi-a-GCST005810	ENSG00000164574 || id:eqtl-a-ENSG00000164574	Inverse-variance weighted (multiplicative random effects)	4	0.307127	0.086586	0	MR Egger	0.612881
SYK	eqtl-a-ENSG00000165025	ebi-a-GCST005810	Osteoarthritis of the hip (hospital-diagnosed) || id:ebi-a-GCST005810	ENSG00000165025 || id:eqtl-a-ENSG00000165025	Inverse-variance weighted (multiplicative random effects)	4	0.041277	0.017723	0.02	MR Egger weighted median	0.826421
NCF1C	eqtl-a-ENSG00000165178	ebi-a-GCST005810	Osteoarthritis of the hip (hospital-diagnosed) || id:ebi-a-GCST005810	ENSG00000165178 || id:eqtl-a-ENSG00000165178	Inverse-variance weighted (multiplicative random effects)	4	−0.11162	0.025492	0	MR Egger weighted median	0.695435
TRANK1	eqtl-a-ENSG00000168016	ebi-a-GCST005810	Osteoarthritis of the hip (hospital-diagnosed) || id:ebi-a-GCST005810	ENSG00000168016 || id:eqtl-a-ENSG00000168016	Inverse-variance weighted (multiplicative random effects)	3	−0.04324	0.019382	0.026	MR Egger weighted median	0.973751
CX3CR1	eqtl-a-ENSG00000168329	ebi-a-GCST005810	Osteoarthritis of the hip (hospital-diagnosed) || id:ebi-a-GCST005810	ENSG00000168329 || id:eqtl-a-ENSG00000168329	Inverse-variance weighted (multiplicative random effects)	5	−0.20561	0.093691	0.028	MR Egger weighted median	0.22481
RAB31	eqtl-a-ENSG00000168461	ebi-a-GCST005810	Osteoarthritis of the hip (hospital-diagnosed) || id:ebi-a-GCST005810	ENSG00000168461 || id:eqtl-a-ENSG00000168461	Inverse-variance weighted (multiplicative random effects)	8	−0.12515	0.06257	0.045	MR Egger weighted median	0.520829
PTAFR	eqtl-a-ENSG00000169403	ebi-a-GCST005810	Osteoarthritis of the hip (hospital-diagnosed) || id:ebi-a-GCST005810	ENSG00000169403 || id:eqtl-a-ENSG00000169403	Inverse-variance weighted (multiplicative random effects)	4	0.269285	0.109184	0.014	MR Egger weighted median	0.535136
NPAS2	eqtl-a-ENSG00000170485	ebi-a-GCST005810	Osteoarthritis of the hip (hospital-diagnosed) || id:ebi-a-GCST005810	ENSG00000170485 || id:eqtl-a-ENSG00000170485	Inverse-variance weighted (multiplicative random effects)	4	0.086253	0.020196	0	MR Egger weighted median	0.827519
PKIA	eqtl-a-ENSG00000171033	ebi-a-GCST005810	Osteoarthritis of the hip (hospital-diagnosed) || id:ebi-a-GCST005810	ENSG00000171033 || id:eqtl-a-ENSG00000171033	Inverse-variance weighted (multiplicative random effects)	10	−0.08369	0.04137	0.043	MR Egger weighted median	0.569548
INSR	eqtl-a-ENSG00000171105	ebi-a-GCST005810	Osteoarthritis of the hip (hospital-diagnosed) || id:ebi-a-GCST005810	ENSG00000171105 || id:eqtl-a-ENSG00000171105	Inverse-variance weighted (multiplicative random effects)	3	0.429477	0.117337	0	MR Egger	0.686507
CEBPB	eqtl-a-ENSG00000172216	ebi-a-GCST005810	Osteoarthritis of the hip (hospital-diagnosed) || id:ebi-a-GCST005810	ENSG00000172216 || id:eqtl-a-ENSG00000172216	Inverse-variance weighted (multiplicative random effects)	4	−0.09969	0.026082	0	MR Egger weighted median	0.851249
DDIT3	eqtl-a-ENSG00000175197	ebi-a-GCST005810	Osteoarthritis of the hip (hospital-diagnosed) || id:ebi-a-GCST005810	ENSG00000175197 || id:eqtl-a-ENSG00000175197	Inverse-variance weighted (multiplicative random effects)	3	0.228411	0.040889	0	MR Egger weighted median	0.875236
MRPL48	eqtl-a-ENSG00000175581	ebi-a-GCST005810	Osteoarthritis of the hip (hospital-diagnosed) || id:ebi-a-GCST005810	ENSG00000175581 || id:eqtl-a-ENSG00000175581	Inverse-variance weighted (multiplicative random effects)	3	0.107887	0.009995	0	MR Egger weighted median	0.906152
CRLF3	eqtl-a-ENSG00000176390	ebi-a-GCST005810	Osteoarthritis of the hip (hospital-diagnosed) || id:ebi-a-GCST005810	ENSG00000176390 || id:eqtl-a-ENSG00000176390	Inverse-variance weighted (multiplicative random effects)	4	0.075777	0.023369	0.001	MR Egger weighted median	0.743505
ADAP2	eqtl-a-ENSG00000184060	ebi-a-GCST005810	Osteoarthritis of the hip (hospital-diagnosed) || id:ebi-a-GCST005810	ENSG00000184060 || id:eqtl-a-ENSG00000184060	Inverse-variance weighted (multiplicative random effects)	3	0.450766	0.217043	0.038	MR Egger weighted median	0.474219
FOXO4	eqtl-a-ENSG00000184481	ebi-a-GCST005810	Osteoarthritis of the hip (hospital-diagnosed) || id:ebi-a-GCST005810	ENSG00000184481 || id:eqtl-a-ENSG00000184481	Inverse-variance weighted (multiplicative random effects)	3	−0.38696	0.034977	0	MR Egger weighted median	0.935967
PDE4B	eqtl-a-ENSG00000184588	ebi-a-GCST005810	Osteoarthritis of the hip (hospital-diagnosed) || id:ebi-a-GCST005810	ENSG00000184588 || id:eqtl-a-ENSG00000184588	Inverse-variance weighted (multiplicative random effects)	3	0.236931	0.041429	0	MR Egger weighted median	0.735599
INSIG1	eqtl-a-ENSG00000186480	ebi-a-GCST005810	Osteoarthritis of the hip (hospital-diagnosed) || id:ebi-a-GCST005810	ENSG00000186480 || id:eqtl-a-ENSG00000186480	Inverse-variance weighted (multiplicative random effects)	7	0.111239	0.043196	0.01	MR Egger weighted median	0.611197
FPR3	eqtl-a-ENSG00000187474	ebi-a-GCST005810	Osteoarthritis of the hip (hospital-diagnosed) || id:ebi-a-GCST005810	ENSG00000187474 || id:eqtl-a-ENSG00000187474	Inverse-variance weighted (multiplicative random effects)	6	−0.21036	0.067108	0.002	MR Egger weighted median	0.703622
CARD9	eqtl-a-ENSG00000187796	ebi-a-GCST005810	Osteoarthritis of the hip (hospital-diagnosed) || id:ebi-a-GCST005810	ENSG00000187796 || id:eqtl-a-ENSG00000187796	Inverse-variance weighted (multiplicative random effects)	6	−0.07716	0.030194	0.011	MR Egger weighted median	0.481819
ATG7	eqtl-a-ENSG00000197548	ebi-a-GCST005810	Osteoarthritis of the hip (hospital-diagnosed) || id:ebi-a-GCST005810	ENSG00000197548 || id:eqtl-a-ENSG00000197548	Inverse-variance weighted (multiplicative random effects)	4	−0.17177	0.070871	0.015	MR Egger weighted median	0.522111
CCDC69	eqtl-a-ENSG00000198624	ebi-a-GCST005810	Osteoarthritis of the hip (hospital-diagnosed) || id:ebi-a-GCST005810	ENSG00000198624 || id:eqtl-a-ENSG00000198624	Inverse-variance weighted (multiplicative random effects)	3	0.20586	0.060369	0.001	MR Egger weighted median	0.863129
HSPA1B	eqtl-a-ENSG00000204388	ebi-a-GCST005810	Osteoarthritis of the hip (hospital-diagnosed) || id:ebi-a-GCST005810	ENSG00000204388 || id:eqtl-a-ENSG00000204388	Inverse-variance weighted (multiplicative random effects)	4	−0.18574	0.048195	0	MR Egger weighted median	0.737545
PPP1CB	eqtl-a-ENSG00000213639	ebi-a-GCST005810	Osteoarthritis of the hip (hospital-diagnosed) || id:ebi-a-GCST005810	ENSG00000213639 || id:eqtl-a-ENSG00000213639	Inverse-variance weighted (multiplicative random effects)	4	0.140212	0.064686	0.03	MR Egger	0.264393
ANG	eqtl-a-ENSG00000214274	ebi-a-GCST005810	Osteoarthritis of the hip (hospital-diagnosed) || id:ebi-a-GCST005810	ENSG00000214274 || id:eqtl-a-ENSG00000214274	Inverse-variance weighted (multiplicative random effects)	9	−0.09934	0.050357	0.049	MR Egger weighted median	0.457848
APOBEC3G	eqtl-a-ENSG00000239713	ebi-a-GCST005810	Osteoarthritis of the hip (hospital-diagnosed) || id:ebi-a-GCST005810	ENSG00000239713 || id:eqtl-a-ENSG00000239713	Inverse-variance weighted (multiplicative random effects)	6	−0.11315	0.026796	0	MR Egger weighted median	0.767311

**Table 2:** IVW model of the CEBPB.

id.Exposure	id.Outcome	Outcome	Exposure	Method	nsnp	b	se	pval	lo_ci	up_ci	or	or_lci95	or_uci95
eqtl-a-ENSG00000172216	ebi-a-GCST005810	Osteoarthritis of the hip (hospital-diagnosed) || id:ebi-a-GCST005810	ENSG00000172216 || id:eqtl-a-ENSG00000172216	MR Egger	4	−0.0239	0.3751	0.9551	−0.75897	0.711265	0.9764	0.468148	2.036566
eqtl-a-ENSG00000172216	ebi-a-GCST005810	Osteoarthritis of the hip (hospital-diagnosed) || id:ebi-a-GCST005810	ENSG00000172216 || id:eqtl-a-ENSG00000172216	Inverse-variance weighted (multiplicative random effects)	4	−0.0997	0.0261	0.0001	−0.15081	−0.04857	0.9051	0.860009	0.952588
eqtl-a-ENSG00000172216	ebi-a-GCST005810	Osteoarthritis of the hip (hospital-diagnosed) || id:ebi-a-GCST005810	ENSG00000172216 || id:eqtl-a-ENSG00000172216	Weighted median	4	−0.1027	0.1244	0.4089	−0.34642	0.141037	0.9024	0.707218	1.151467
eqtl-a-ENSG00000172216	ebi-a-GCST005810	Osteoarthritis of the hip (hospital-diagnosed) || id:ebi-a-GCST005810	ENSG00000172216 || id:eqtl-a-ENSG00000172216	Simple mode	4	−0.1323	0.167	0.486	−0.45961	0.194957	0.8761	0.631528	1.215259
eqtl-a-ENSG00000172216	ebi-a-GCST005810	Osteoarthritis of the hip (hospital-diagnosed) || id:ebi-a-GCST005810	ENSG00000172216 || id:eqtl-a-ENSG00000172216	Weighted mode	4	−0.1075	0.1456	0.5138	−0.39285	0.177832	0.8981	0.675133	1.194625

**Table 3:** IVW model of CX3CR1.

id.Exposure	id.Outcome	Outcome	Exposure	Method	nsnp	b	se	pval	lo_ci	up_ci	or	or_lci95	or_uci95
eqtl-a-ENSG00000168329	ebi-a-GCST005810	Osteoarthritis of the hip (hospital-diagnosed) || id:ebi-a-GCST005810	ENSG00000168329 || id:eqtl-a-ENSG00000168329	MR Egger	5	−0.396	0.153	0.0812	−0.6959	−0.09612	0.673	0.498624	0.908354
eqtl-a-ENSG00000168329	ebi-a-GCST005810	Osteoarthritis of the hip (hospital-diagnosed) || id:ebi-a-GCST005810	ENSG00000168329 || id:eqtl-a-ENSG00000168329	Inverse-variance weighted (multiplicative random effects)	5	−0.2056	0.0937	0.0282	−0.38925	−0.02198	0.8141	0.677567	0.978261
eqtl-a-ENSG00000168329	ebi-a-GCST005810	Osteoarthritis of the hip (hospital-diagnosed) || id:ebi-a-GCST005810	ENSG00000168329 || id:eqtl-a-ENSG00000168329	Weighted median	5	−0.2015	0.1042	0.0531	−0.4058	0.002726	0.8175	0.666446	1.002729
eqtl-a-ENSG00000168329	ebi-a-GCST005810	Osteoarthritis of the hip (hospital-diagnosed) || id:ebi-a-GCST005810	ENSG00000168329 || id:eqtl-a-ENSG00000168329	Simple mode	5	−0.1856	0.1347	0.2405	−0.44964	0.078536	0.8306	0.637857	1.081703
eqtl-a-ENSG00000168329	ebi-a-GCST005810	Osteoarthritis of the hip (hospital-diagnosed) || id:ebi-a-GCST005810	ENSG00000168329 || id:eqtl-a-ENSG00000168329	Weighted mode	5	−0.221	0.1248	0.1512	−0.46556	0.023509	0.8017	0.627786	1.023788

**Table 4:** Sensitivity analysis.

Gene	id.Exposure	id.Outcome	Outcome	Exposure	Method	Q	Q_df	Q_pval
CEBPB	eqtl-a-ENSG00000172216	ebi-a-GCST005810	Osteoarthritis of the hip (hospital-diagnosed) || id:ebi-a-GCST005810	ENSG00000172216 || id:eqtl-a-ENSG00000172216	MR Egger	0.1051	2	0.9488
CEBPB	eqtl-a-ENSG00000172216	ebi-a-GCST005810	Osteoarthritis of the hip (hospital-diagnosed) || id:ebi-a-GCST005810	ENSG00000172216 || id:eqtl-a-ENSG00000172216	Inverse-variance weighted	0.1503	3	0.9852
CX3CR1	eqtl-a-ENSG00000168329	ebi-a-GCST005810	Osteoarthritis of the hip (hospital-diagnosed) || id:ebi-a-GCST005810	ENSG00000168329 || id:eqtl-a-ENSG00000168329	MR Egger	2.1718	3	0.5375
CX3CR1	eqtl-a-ENSG00000168329	ebi-a-GCST005810	Osteoarthritis of the hip (hospital-diagnosed) || id:ebi-a-GCST005810	ENSG00000168329 || id:eqtl-a-ENSG00000168329	Inverse-variance weighted	4.4956	4	0.3431

**Table 5:** Horizontal pleiotropy test.

Gene	id.Exposure	id.Outcome	Outcome	Exposure	Egger_Intercept	se	pval
CEBPB	eqtl-a-ENSG00000172216	ebi-a-GCST005810	Osteoarthritis of the hip (hospital-diagnosed) || id:ebi-a-GCST005810	ENSG00000172216 || id:eqtl-a-ENSG00000172216	−0.0135	0.0636	0.8512
CX3CR1	eqtl-a-ENSG00000168329	ebi-a-GCST005810	Osteoarthritis of the hip (hospital-diagnosed) || id:ebi-a-GCST005810	ENSG00000168329 || id:eqtl-a-ENSG00000168329	0.051	0.0335	0.2248

## Data Availability

The original contributions presented in this study are included in the article/supplementary material. Further inquiries can be directed to the corresponding authors.

## Supplementary Materials

The following supporting information can be downloaded at: https://www.mdpi.com/article/10.3390/bioengineering12090930/s1, Table S1: IV screening of CEBPB and CX3CR1.
