# Supplementary material for: Mendelian Randomization and Transcriptome Analyses Reveal Important Roles for CEBPB and CX3CR1 in Osteoarthritis
Source: Bioengineering (Basel). 2025 Aug 29;12(9):930. doi: 10.3390/bioengineering12090930 (PMC12467285; doi:10.3390/bioengineering12090930)
Supplement: Supplementary file 1 [file bioengineering-12-00930-s001.zip › Table S1.pdf]

Table S1. IV screening of CEBPB and CX3CR1

gene	exposure	outcome	id.exposure	id.outcome	sam-plesize	SNP	b	se	p
CEBPB	ENSG00000172216 || id:eqtl-a-ENSG00000172216	Osteoarthritis of the hip (hospital diagnosed) || id:ebi-a-GCST005810	eqtl-a-ENSG00000172216	ebi-a-GCST005810	11989	rs149110519	-0.00817	0.549587	0.988136
CEBPB	ENSG00000172216 || id:eqtl-a-ENSG00000172216	Osteoarthritis of the hip (hospital diagnosed) || id:ebi-a-GCST005810	eqtl-a-ENSG00000172216	ebi-a-GCST005810	11989	rs1889236	-0.05883	0.177709	0.740598
CEBPB	ENSG00000172216 || id:eqtl-a-ENSG00000172216	Osteoarthritis of the hip (hospital diagnosed) || id:ebi-a-GCST005810	eqtl-a-ENSG00000172216	ebi-a-GCST005810	11989	rs7210990	-0.15977	0.345224	0.643515
CEBPB	ENSG00000172216 || id:eqtl-a-ENSG00000172216	Osteoarthritis of the hip (hospital diagnosed) || id:ebi-a-GCST005810	eqtl-a-ENSG00000172216	ebi-a-GCST005810	11989	rs913678	-0.13577	0.181711	0.454941
CEBPB	ENSG00000172216 || id:eqtl-a-ENSG00000172216	Osteoarthritis of the hip (hospital diagnosed) || id:ebi-a-GCST005810	eqtl-a-ENSG00000172216	ebi-a-GCST005810	11989	All - Inverse variance weighted (multiplicative random effects)	-0.09969	0.026082	0.000132
CEBPB	ENSG00000172216 || id:eqtl-a-ENSG00000172216	Osteoarthritis of the hip (hospital diagnosed) || id:ebi-a-GCST005810	eqtl-a-ENSG00000172216	ebi-a-GCST005810	11989	All - MR Egger	-0.02385	0.37506	0.955075
CX3CR1	ENSG00000168329 || id:eqtl-a-ENSG00000168329	Osteoarthritis of the hip (hospital diagnosed) || id:ebi-a-GCST005810	eqtl-a-ENSG00000168329	ebi-a-GCST005810	11989	rs11711752	-0.48041	0.201611	0.017178
CX3CR1	ENSG00000168329 || id:eqtl-a-ENSG00000168329	Osteoarthritis of the hip (hospital diagnosed) || id:ebi-a-GCST005810	eqtl-a-ENSG00000168329	ebi-a-GCST005810	11989	rs1354034	0.318191	0.411892	0.439813
CX3CR1	ENSG00000168329 || id:eqtl-a-ENSG00000168329	Osteoarthritis of the hip (hospital diagnosed) || id:ebi-a-GCST005810	eqtl-a-ENSG00000168329	ebi-a-GCST005810	11989	rs55908509	0.121539	0.327433	0.710499
CX3CR1	ENSG00000168329 || id:eqtl-a-ENSG00000168329	Osteoarthritis of the hip (hospital diagnosed) || id:ebi-a-GCST005810	eqtl-a-ENSG00000168329	ebi-a-GCST005810	11989	rs7651994	-0.20884	0.131275	0.111648

CX3CR1	ENSG00000168329 || id:eqtl-a-ENSG00000168329	Osteoarthritis of the hip (hospital diagnosed) || id:ebi-a-GCST005810	eqtl-a-ENSG00000168329	ebi-a-GCST005810	11989	rs79589879	-0.17882	0.182013	0.325879
CX3CR1	ENSG00000168329 || id:eqtl-a-ENSG00000168329	Osteoarthritis of the hip (hospital diagnosed) || id:ebi-a-GCST005810	eqtl-a-ENSG00000168329	ebi-a-GCST005810	11989	All - Inverse variance weighted (multiplicative random effects)	-0.20561	0.093691	0.028193
CX3CR1	ENSG00000168329 || id:eqtl-a-ENSG00000168329	Osteoarthritis of the hip (hospital diagnosed) || id:ebi-a-GCST005810	eqtl-a-ENSG00000168329	ebi-a-GCST005810	11989	All - MR Egger	-0.39601	0.153006	0.081199
